# “Il flauto magico” still works: Mozart’s secret of ventilation

**DOI:** 10.1186/2049-6958-8-23

**Published:** 2013-03-19

**Authors:** Klaus Laczika, Oliver P Graber, Gerhard Tucek, Alfred Lohninger, Nikolaus Fliri, Gertraud Berka-Schmid, Eva K Masel, Christoph C Zielinski

**Affiliations:** 1 Medical University of Vienna, Department of Internal Medicine 1, Division of Palliative Care, Waehringer Guertel 18-20, Vienna 1090, Austria; 2 University of Music and Performing Arts Vienna, Institute 13 for Music and Movement Education, and Music Therapy, Rennweg 8, Vienna, 1030, Austria; 3 IMC University of Applied Sciences, Piaristengasse 1, Krems, 3500, Austria; 4 Autonom Health, Cobenzlgasse 74-76/Top 1, Vienna, 1190, Austria

**Keywords:** Breathing patterns, Mozart, Synchronisation of biological and musical rhythms, Mozar

## Abstract

**Background:**

Synchronisation/coupling between respiratory patterns and musical structure.

**Methods:**

Healthy professional musicians and members of the audience were studied during a performance of W.A. Mozart’s Piano Concerto KV 449. Electrocardiogram (ECG)/Heart Rate Variability (HRV) data recording (Schiller: Medilog^®^AR12, ECG-channels: 3, sampling rate: 4096 Hz, 16 Bit) was carried out and a simultaneous synchronized high definition video/audio recording was made. The breathing-specific data were subsequently extracted using Electrocardiogram-derived respiration (EDR; Software: Schiller medilog^®^DARWIN) from the HRV data and overlaid at the same time onto the musical score using FINALE 2011 notation software and the GIMP 2.0 graphics programme. The musical score was graphically modified graphically so that the time code of the breathing signals coincided exactly with the notated musical elements. Thus a direct relationship could be produced between the musicians’ breathing activity and the musical texture. In parallel with the medical/technical analysis, a music analysis of the score was conducted with regard to the style and formal shaping of the composition.

**Results:**

It was found that there are two archetypes of ideally typical breathing behaviour in professional musicians that either drive the musical creation, performance and experience or are driven by the musical structure itself. These archetypes also give rise to various states of synchronisation and regulation between performers, audience and the musical structure.

**Conclusions:**

There are two archetypes of musically-induced breathing which not only represent the identity of music and human physiology but also offer new approaches for multidisciplinary respiratory medicine.

## Background

Music is breathing: phrasing is nothing other than a “mirror” of the lung’s activity. Without the optimal use of ventilation (a very special ability of professional musicians requiring years of training) musical performance, and even the composition of music, become impossible for the reason that musical rhythms and biological rhythms – especially breathing/breathing patterns – are strongly related to each other.

Especially recently a multitude of studies focused on various aspects of different musically- induced effects upon human physiology. In addition to approaches from neuroscience, which are pursued for the most part using functional imaging (fMRI, PET, etc; an overview is given in, e.g. [1-3]), a central interest has been musically-induced physiological regulatory processes regarding the autonomic nervous system [4] or the cardio-vascular and respiratory system [5-12].

With only a few exceptions [12], which are at best rudimentary in their approach [13,14], the investigations known to us concerning this matter surprisingly omit any theoretical musical analysis of the stimuli employed. In this way a very one-sided and incomplete gain in knowledge arises that does not allow detailed conclusions to be drawn concerning the effects of musical (micro) structures on the reached conclusions.

Moreover, what is missing is how far empirical observation informs the art of musical composition and gives guidance regarding how music can be optimised in order to realize a relationship towards listeners. (“*Mein Fürst war mit allen meinen Arbeiten zufrieden*, *ich erhielt Beyfall*, *ich konnte als Chef eines Orchesters Versuche machen*, *beobachten*, *was den Eindruck hervorbringt*, *und was ihn schwächt*, *also verbessern*, *zusetzen*, *wegschneiden*, *wagen*. *Ich war von der Welt abgesondert*, *niemand in meiner Nähe konnte mich an mir selber irre machen und quälen*, *und so mußte ich original werden*.” – “*My Prince was happy with all my work*, *I was applauded*, *as the conductor of an orchestra I could experiment*, *observe what creates an impression and what weakens it*, *and thus improve*, *add*, *take away*, *dare*; *I was cut off from the world*, *nobody near me could confuse and torment me*, *and so I had to become original*.” Joseph Haydn, quoted according to [15]. In so doing so physiological aspects also influence the musical structure: in particular, these are adaptations of the musical structure to the needs of the recognition of particular elements, the perception of musical structure itself, memory in general [16-18] and numerous papers building on these as well as the (total) duration, breathing, and tempo [19-21]. In this regard theoretical textbooks on music partly refer directly to physiological terminology [19,22-25] or delineate the possibility of a therapeutic use of music [20]. The situation is similar in the field of musical interpretation, in which, already within the framework of training, great value is set on conscious optimisation of the physiological parameters (breathing, muscle tension…) of musical reproduction in order to actively control them [26-30].

As physiological parameters are thus directly anchored and present in musical structure and furthermore enter actively and consciously into the process of interpretation, account must be taken of the fact that the investigation of the interaction of music and the human organism in most cases amounts in the end to the analysis of interactions of two “biological systems” per se. (excluded from this are musical structures which, for example, were generated in a purely stochastic way or originate from serial concepts, etc.).

## Methods

Healthy professional musicians (string players and a pianist) and healthy members of the audience were studied during a public performance of Wolfgang Amadeus Mozart’s Piano Concerto No. 14, KV 449, version for string orchestra and piano: 11 healthy professional male musicians, age 35–67 (10 string players and members of the Vienna Philharmonic Orchestra, 1 healthy soloist/pianist, age 47) and 12 (8 male/4 female, age: 42–68) members of the audience. Electrocardiogram (ECG)/Heart Rate Variability (HRV) data recording (Schiller: Medilog^®^AR12, ECG-channels: 3, sampling rate: 4096 Hz, 16 Bit) was carried out while at the same time a synchronized high definition video/audio recording was made. The breathing-specific data were subsequently extracted using Electrocardiogram-derived respiration (EDR; Software: Schiller Medilog^®^DARWIN) from the HRV data and overlaid at the same time onto the musical score using FINALE 2011 notation software and the GIMP 2.0 graphics programme. The musical score was modified graphically so that the time code of the breathing signals coincided exactly with the notated musical elements. Thus a direct relationship could be produced between the musicians’ breathing activity and the musical texture. In parallel with the medical/technical analysis, a formal musical analysis of the score was conducted with regard to the style and structure of the composition.

## Results and discussion

It was found that there are two archetypes of ideally typical breathing behaviour in professional musicians that either drive the musical creation, performance, and experience or are driven by the musical structure itself. These archetypes also give rise to various states of synchronisation and regulation between performers, audience and the music. Based on the assumptions that music is breathing and Mozart’s music represents the phrasing (in other words, musical breathing) in an ideal way, the score of his Piano Concerto KV 449 makes breathing not only appreciable and apparent but also brings breathing patterns into the focus of attention for both musicians and the audience. Based upon this collectively perceptible “sonificated ventilography” the coupling of music and physiology can start to take place.

In the direct comparison of the musical structure and the breathing activity, ideally typical patterns appear corresponding to a direct “bilateral influence” in the conjunction of music and physiology.

**Figure 1:**
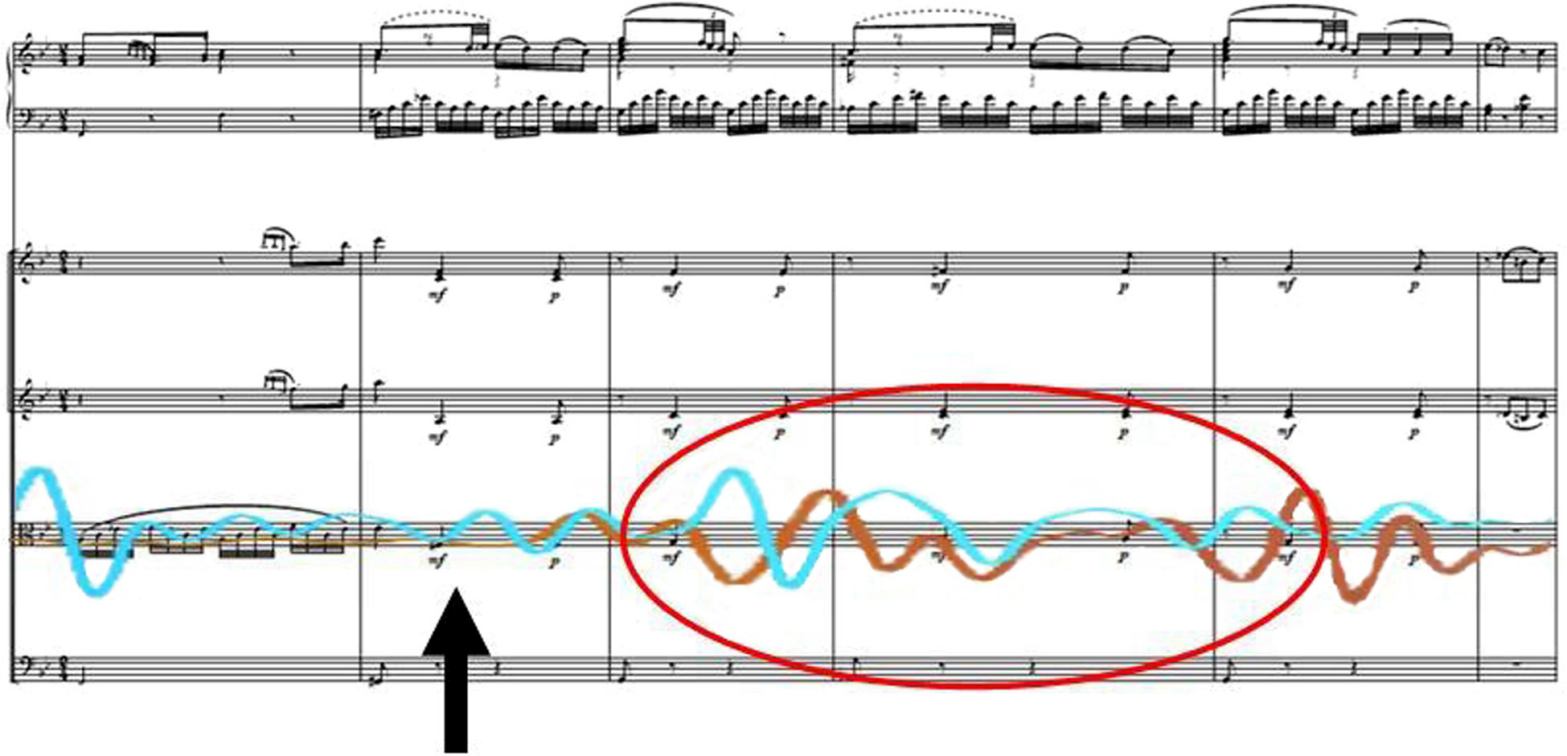
**Active musically-shaped breathing and passively musically-shaped breathing exemplified by the violas (circled in red). **Arrow: “Start point” of the process at the syncopation; bars 44–49.

The example of two viola players playing at one desk Figure 1 demonstrates that their breathing on the one hand is appropriate to the instrumental playing (and thus corresponds simultaneously to its requirements). On the other hand, however, this adaptation and control in relation to instrumental playing can also be inhibited in order to respond to other involved musicians as a “foreign stimulus” (in the sense of resonance and synchronisation phenomena).

Therefore we designate these two types of breathing as

I. **Active musically-shaped breathing**

II. **Passive musically-shaped breathing.**

**Figure 2:**
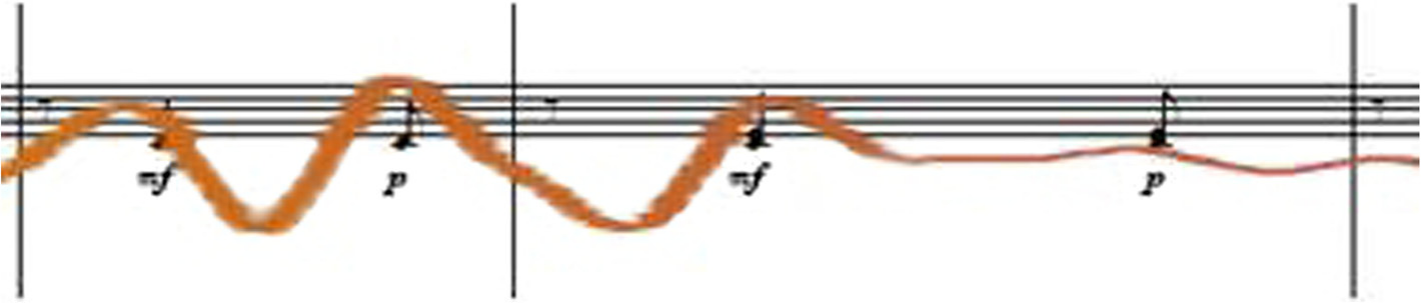
**Ideally typical *sforzati *breathing in the example of the 2nd violin bars 46/47; clef: treble clef.**

The brown line of Figure 1 represents a type of breathing that is developed by professional music training. It, therefore, conforms above all to the course of the *sforzati* and hence the syncopations. This is also ideally and typically represented in the example of the second violin Figure 2, likewise coloured in brown].

As it is also known and described in Martial Arts [31], the consciously-shaped course of the breathing during instrumental playing serves not only the phrasing, but also the maximising of the gain in force (powerful expiration). It is for this reason that dynamic accents are linked over all the instrument groups with a motion of expiration. This strategy is also a central issue of professional musical education and training. Moreover, musicians possess a profound knowledge regarding the central role of the musical upbeat, which forms a clear and well- defined inspiration, and they are usually taught to use their respiration as an aid to master metrical difficulties (so called counting music with the body).

**Figure 3:**
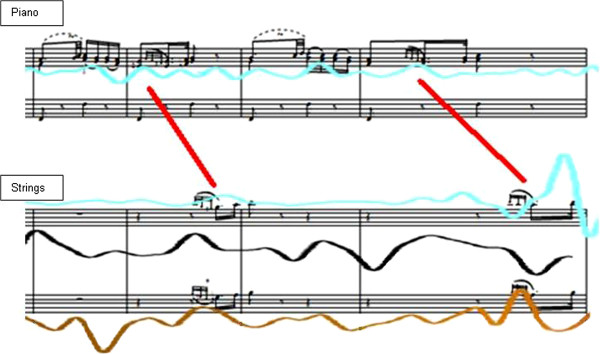
**Question and answer breathing between solo piano and upper strings in bars 41–44.** Clefs: piano upper stave, treble clef; lower stave, bass clef; strings: both staves treble clef. The accented notes for the production of the grace notes are clearly discernible, the 2^nd ^player at the leader’s desk (shown in black) changes at the same time at bar 44 from an active musically-shaped breathing to the passively musically-shaped breathing, while his breath line adapts to the events in the solo piano part.

This up-beat can be represented (by reason of the musical structure) as a dialogue and responsorial event in the sense of “question and answer breathing”, e.g. in bar 42 and in the corresponding places in bars 44 and 46) in comparison with the data of the soloist with the upper strings Figure 3).

Thus far the “synchronisations” found for example in our study between elements appearing in the score and patterns of breathing correlating to them are to be considered simply as evidence of the instrumental professionalism of the performers and thus as neither surprising nor accidental. Beyond that, however, they illustrate how closely physiological parameters are connected with musical structure/elements per se, and we would like to point out once again that this is obviously also of significance for the shaping of the composition. Thus, it is a fundamental question in the area of instrumentation how much breath a specific instrument needs in general in order to execute tone groups and how much time, for example, slurs may require as a result in relation to the stipulated sound volume and register [32-37].

**Figure 4:**
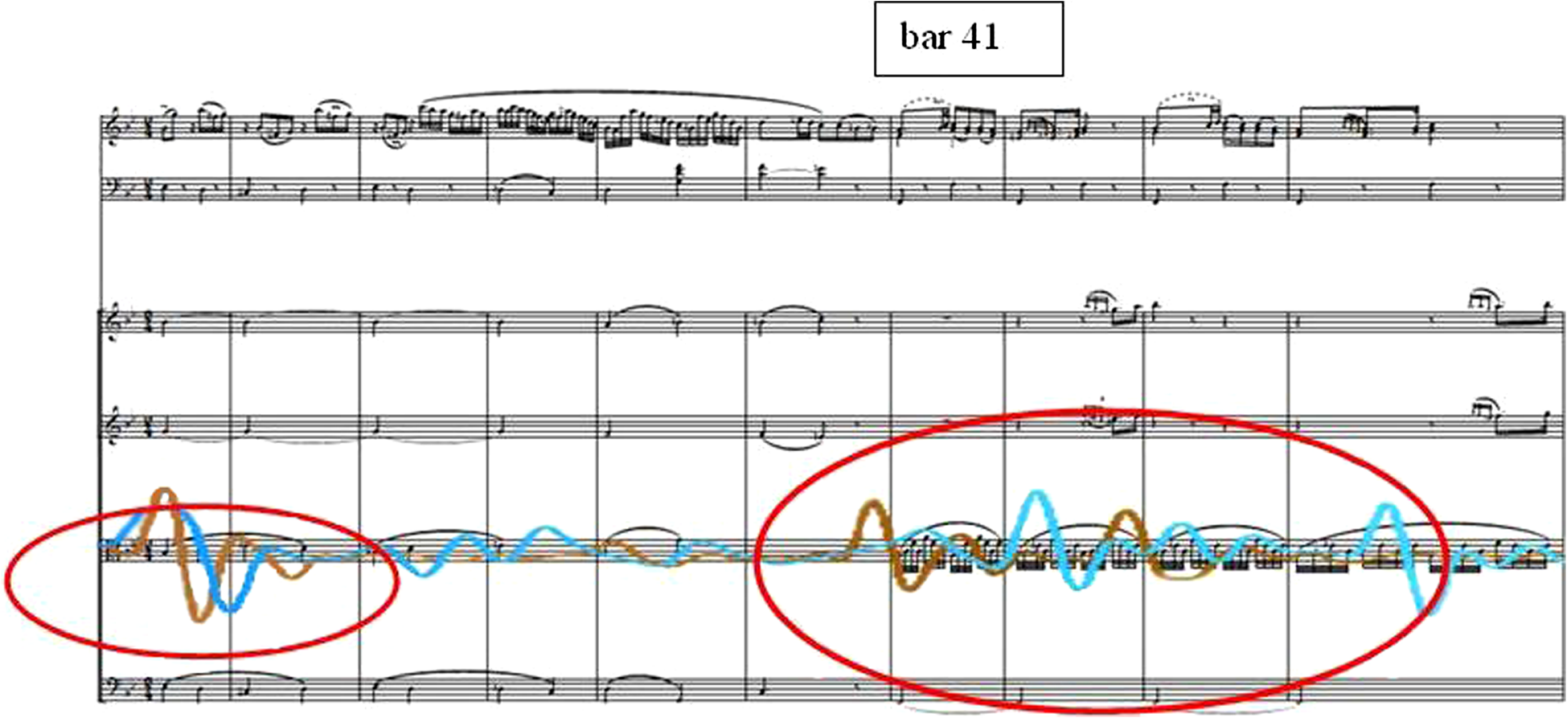
The breath patterns of the viola players further compared.

The blue line in Figure 1 above shows the breathing “uncoupled” from the musician`s own playing and its needs, and detached by virtue of attentive listening orientated to the playing of the soloist. The breathing is adapted passively to the grace notes of the piano and the time distortions accompanying them (in the sense of a lengthening and shortening) by the beat, which is altered in time and by amplitude. The solo piano works in this sense as a “disturbance” of the breath pattern planned by the strings. It is conspicuous that this pattern also appears for this musician at corresponding places in the musical form and sands out clearly from the breathing appropriate purely to the playing needs of the second viola player where it also dispenses with up-beat breathing in favour of following the score (as we could already observe in Figure 3 bar 44 with the other musician Figure 4.

**Figure 5:**
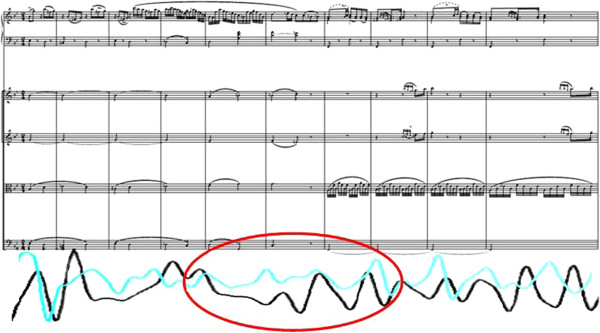
Transition from “metrically uncontrolled” to “metrically controlled” breathing by members of the audience in the sense of a reaction to Mozart’s play on expectations and structure formation.

These striking features of the breath-shaping (including a clearly discernible “preparation” at bars 41 ff. Figure 5 at a musically and structurally relevant place in the course of the movement) as well as the frequency of its appearance and point in time of their manifestation also correlate formally with the results of the study based on musical analysis, as the musical process in bars 44 ff. (beginning of the subsidiary figure, ger: “*Seitengedanke*”) is given prominence by many aspects in the course of the work and movement Figure 5.

One of the new musical elements coming in at this point of formal development is the 1/32 subdivision of the beat, movement, which leads to the climax of tension in bar 45 and thus underlines the harmony.

The features of the composition in bar 4 includes can be summarized in the following way:

Bar 41:

•Marks the beginning of the second subject group

•Captures the hitherto deepest sound range of the 2^nd^ movement

•Is the beginning of a pedal point

•Brings into play for the first time in this movement the 1/32 (demisemiquaver) subdivision of the beat (in style of an Alberti bass)

•Exposes a clear two-bar structure as it develops

•Is the start of a short sequence chain (above the pedal point)

•Presents, as it develops, echo effects and question and answer scenarios” in the interplay between solo piano and different interjections from by the upper strings

Harmonic analysis of bars 41–44 (pedal point):

**Opening key of the movement: Bb major** (= I^st^ degree/tonic)

**Bar 41** Pedal point F natural, above it F major (= V^th^ degree/dominant of the opening key of the movement)

**Bar 42** Pedal point F natural, above it V^7^/dominant of F major, the F sharp in the grace notes makes the dominant of the dominant (=G major) sound like a chromatic auxiliary note which is foreign to the key.

**Bar 43** Pedal point F natural, above it the same harmonic situation as in bar 42 (now, however, without the “F sharp” and thus without the double dominant appeal of the grace notes).

**Bar 44** Pedal point F natural, above it V^7^, that is the dominant 7 of Bb major [as the seventh chord on the tonic of F major with flattened 7^th^, the E natural (grace note) of the high strings still points to F major and thus brings to mind both of the following: a) Root of V^7^/D^7^ in Bb major and b) Root of F major as the dominant key of Bb major.

**Figure 6:**
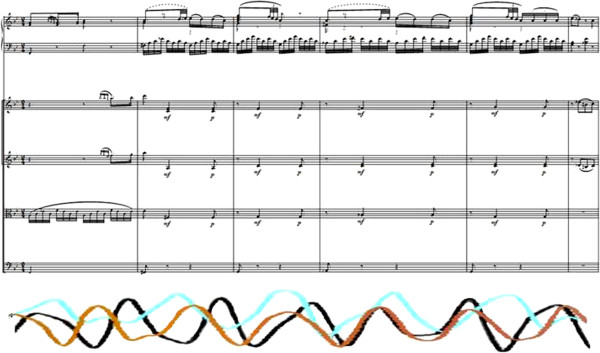
Breath pattern of 3 listeners (overlaid) as an example of a whole-bar synchronising exhalation.

The synchronisation processes of the breath activity for the listeners show themselves to be in accordance with the findings of Haas et al. [8] as whole-bar-breathing, which can be attributed to the basic compositional structure and the musical shaping. In the course of this breathing in the “pulse” of the composition, there was also a corresponding heightening for the listeners in HRV (heart rate variability) in the sense of a vagotonus. In relation to the special position of bar 41 already described above, significant alterations in the HRV can also be described which, in accordance with the musical analysis, can be seen as standing directly in correlation with musical expectation and the formal and structural course of the movement Figure 6.

The results make two archetypes of breathing and breath technique by professional musicians comprehensible, which on the one hand serve the technical playing and interpretational requirements – and with them the compositional structure of the movements – and on the other are to be considered as an expression of the adaptive experiencing of the mutual music making situation. In this situation it becomes clear that such correlations can only be shown under a detailed consideration of the musical text and a corresponding graphic reappraisal in regard to the music theoretic analysis. To a large extent they escape a statistical capture for the reason that, in relation to one and the same “musical stimulus“ they tend to highly meaningful but likewise contrary patterns. The question freshly raised again and again, of the comparison of active music making and passive music listening, which was also evaluated recently for the group of professional musicians [38], is supported through our study as it was in previous assumptions - also in relation to the breath models of the audience. It is enriched by one new fact, in that our findings point to professional musicians tending either to “active” leading or “passive” following during their playing, and that this attitude can also completely change, accompanied by correspondingly measurable alterations to their physiological configuration. Also, the relationship currently being discussed between motor components and melody shaping [39] goes back in the end to the breath action, where the relationship between breathing, breathing action and phrasing has been described in music theory since *Archytas* from Tarent and is handed down above all unnumbered numerous papers on counterpoint. In this respect the present study is a further piece of evidence for these close relationships and is thus suited to be taken up by all specialist disciplines involved as material for further studies. Above all, however, far beyond any ideological attitude, it underlines the necessity of considering music as a “single entity”, of which account is to be taken in an appropriate way in any analysis. An essential aspect of compositional will is in the end based on this attribute, so that the practice of art represents something, in which “*every case* [i.e. every work] *is a new one*, *everyone an exception*” [40]. The so called “*Mozart effect*” [41] may remain controversial, whereas Mozart’s impact on respiration is incontestable.

## Conclusion

Mozart’s drug still exerts its effect more than two centuries later: the two archetypes of ideally typical breathing behaviour /breathing patterns in professional musicians can only be revealed and understood as long as the underlying musical structure provides the coordinates of time which keeps some kind of synchronisation of weakly coupled oscillators (music on the one hand and physiology on the other) ticking over. Additionally the still thrilling influence of Mozart’s latent breathing patterns on today’s audience represents another coupling mechanism of breathing synchronisation between musicians and their listeners. These findings might be useful for respiratory medicine, musical composition, performance, and the development of new interdisciplinary therapeutic concepts. In general musical structure is more deeply interwoven with physiology than has hitherto been scientifically accepted.

## Competing interests

The authors declare that they have no competing/conflicting interests.

## Authors’ information

The HD recording of the underlying performance has been deposited on youtube: http://www.youtube.com/watch?v=tr1m9UnjvlM.
